# Evidence of Positive Selection of Aquaporins Genes from *Pontoporia blainvillei* during the Evolutionary Process of Cetaceans

**DOI:** 10.1371/journal.pone.0134516

**Published:** 2015-07-30

**Authors:** Simone Lima São Pedro, João Marcelo Pereira Alves, André Silva Barreto, André Oliveira de Souza Lima

**Affiliations:** 1 Laboratório de Genética Molecular, Centro de Ciências Tecnológicas da Terra e do Mar, Universidade do Vale do Itajaí, Itajaí, SC, Brazil; 2 Departamento de Parasitologia, Instituto de Ciências Biomédicas, Universidade de São Paulo, São Paulo, SP, Brazil; 3 Laboratório de Informática da Biodiversidade e Geomática, Centro de Ciências Tecnológicas da Terra e do Mar, Universidade do Vale do Itajaí, Itajaí, SC, Brazil; University of Bari Aldo Moro, ITALY

## Abstract

**Background:**

Marine mammals are well adapted to their hyperosmotic environment. Several morphological and physiological adaptations for water conservation and salt excretion are known to be present in cetaceans, being responsible for regulating salt balance. However, most previous studies have focused on the unique renal physiology of marine mammals, but the molecular bases of these mechanisms remain poorly explored. Many genes have been identified to be involved in osmotic regulation, including the aquaporins. Considering that aquaporin genes were potentially subject to strong selective pressure, the aim of this study was to analyze the molecular evolution of seven aquaporin genes (AQP1, AQP2, AQP3, AQP4, AQP6, AQP7, and AQP9) comparing the lineages of cetaceans and terrestrial mammals.

**Results:**

Our results demonstrated strong positive selection in cetacean-specific lineages acting only in the gene for AQP2 (amino acids 23, 83, 107,179, 180, 181, 182), whereas no selection was observed in terrestrial mammalian lineages. We also analyzed the changes in the 3D structure of the aquaporin 2 protein. Signs of strong positive selection in AQP2 sites 179, 180, 181, and 182 were unexpectedly identified only in the baiji lineage, which was the only river dolphin examined in this study. Positive selection in aquaporins AQP1 (45), AQP4 (74), AQP7 (342, 343, 356) was detected in cetaceans and artiodactyls, suggesting that these events are not related to maintaining water and electrolyte homeostasis in seawater.

**Conclusions:**

Our results suggest that the AQP2 gene might reflect different selective pressures in maintaining water balance in cetaceans, contributing to the passage from the terrestrial environment to the aquatic. Further studies are necessary, especially those including other freshwater dolphins, who exhibit osmoregulatory mechanisms different from those of marine cetaceans for the same essential task of maintaining serum electrolyte balance.

## Introduction

The transition from the terrestrial to the aquatic environment occurred in the evolutionary process of cetaceans, resulting in notable and distinct morphological and physiological changes, fundamental to survival in an exclusively aquatic environment [1,2,3,4,5]. But one of the most critical and key changes was adaptation to a highly saline environment. The vast majority of cetaceans are hypoosmotic against seawater, i.e, there is a tendency to lose water naturally by osmosis, leaving them in constant risk of dehydration. To deal with this problem, several strategies are employed to increase water retention in the body [4,6].

Morphological and physiological evidence has been described that is related to mechanisms that enable cetaceans to survive in a hyperosmotic environment such as seawater and still maintain internal homeostasis. Morphological characteristics of the kidneys, such as a relatively large size when compared with those of other mammals, and hundreds of individual lobes, or reniculi [7,8,9], are associated with the ability to retain water. They also exhibit increased medullary thickness, necessary to produce a highly concentrated urine, resulting in excretion of the excess salt from food and ingested seawater, therefore reducing water loss [4,10,11,12]. The urinary electrolyte levels are likely to reflect their food intake to a large extent, since these animals have neither sweat glands nor specialized glands to excrete salt [11].

Cetaceans acquire fresh water via food consumption, water derived from metabolism, and by drinking seawater [4,11]. However, the fact that cetaceans drink seawater is still controversial. Hui [2] demonstrated the irrelevance of drinking seawater, since the metabolic water should be sufficient to maintain water balance. However, Costa [11] reports that cetaceans ingest seawater in cases of stress, with extensive long fasting periods associated with natural water loss through skin, urine, and feces and thus cetaceans would use seawater as a mechanism to maintain osmotic balance. The physiological, anatomical, and behavioral strategies developed by the group allow them to cope with a hyperosmotic environment, but the molecular bases underlying these mechanisms remain poorly explored.

Living cells need to exchange water constantly with their environment and the passage of water from a less concentrated medium to a more concentrated–osmosis–is a natural pre-requisite for life, representing one of the crucial factors leading to cellular homeostasis. For many years, water was considered to enter and leave cells exclusively through the lipid membrane, but the discovery of proteins called aquaporins, which acted as pores or water channels, was a great revolution in research [5,13]. Aquaporins (AQPs) are a family of very hydrophobic small integral plasma membrane proteins, present in all forms of life including mammals, amphibians, insects, plants, and bacteria [14,15,16,17]. AQPs have six transmembrane domains with five connecting loops, three on the extracellular side and two on the intracellular side, with both N- and C- termini facing the cytosol. Two loops dip into the membrane from opposite sides and fold back as half helices making up a seventh pseudo helix, to form the pore that is selective to water and/or solutes, where there are two highly conserved asparagine-proline-alanine (NPA) residues [16,18,19]. Three-dimensional (3D) structural analyses of AQPs have revealed a uniform structure: a tetramer with a pore in each subunit [19].

These proteins have been identified and characterized in various organs directly involved in the transport of large volumes of water and small solutes across cell membranes, such as the kidneys, that need to precisely and/or quickly respond to osmotic conditions of the environment. The AQPs present in the plasma membrane have a 5 to 50-fold higher osmotic water permeability than those in other locations [20]. The aquaporins in mammals are divided into three subgroups: aquaporins with water-specific channels (AQP0, AQP1, AQP2, AQP4, AQP5, AQP6, AQP8), aquaglyceroporins that conduct small neutral solutes like glycerol and urea in addition to polar water molecules channels (AQP3, AQP7, AQP9) [3,19,21,22], and a new subfamily, the 'superaquaporins’ (AQP11, AQP12) [16,23]. Considering aquaporin genes to be subjected to potentially strong selective pressure as previously reported by Ishibashi et al. [19] and Xu et al. [12], the aim of this study was to analyze the molecular evolution of seven aquaporins (AQP1, AQP2, AQP3, AQP4, AQP6, AQP7 and AQP9) identified in *Pontoporia blainvillei* and other cetaceans in comparison with terrestrial mammals in order to investigate what changes might have occurred in the aquaporin genes during the passage of the terrestrial to the aquatic environment.

*Pontoporia blainvillei* is a small cetacean endemic to coastal waters of South America, from northern Argentina to southeastern Brazil [24]. It is considered as “vulnerable” both on its full range [25] and for the specific subpopulation of Rio Grande do Sul/Uruguay [26], mostly because of high rates of incidental capture in gillnets. This species is particularly interesting to study when trying to understand the evolution of water regulation systems because it is the only ‘river dolphin’ that actually inhabits a marine environment. It has been hypothesized [27] that its ancestors colonized the shallow epicontinental seas that inundated the Parana Basin and diversified within its complex fluvial-estuarine-marine system. With the retreat of the marine waters from the Parana basin, *Pontoporia* colonized the nearshore coastal areas north and south of the La Plata estuary, which it inhabits at present. Therefore it is probable that during its evolutionary history the species had to cope with different levels of salinity and thus to change its water balance.

## Materials and Methods

Based in our preliminary draft genome sequencing effort of *Pontoporia blainvillei*, seven aquaporins (AQPs) were described—AQP1, AQP2, AQP3, AQP4, AQP6, AQP7, and AQP9. All new coding sequences determined have been deposited in GenBank under accession numbers KM888076 to KM888082. Orthologous sequences to these aquaporins were obtained from the NCBI database. A total of 33 mammalian species, including 10 orders, were analyzed in this study, namely Primates, Rodentia, Lagomorpha, Dasyuromorphia, Proboscidea, Carnivora, Chiroptera, Artiodactyla, Perissodactyla, and Cetacea (*Tursiops truncatus*, *Lipotes vexillifer*, *Delphinapterus leucas*, *Sousa chinensis*, *Neophocaena phocaenoides*, *Mesoplodon densirostris*, *Balaenoptera acutorostrata scammoni*, *Physeter catodon*, *Pontoporia blainvillei*). Three non-mammalian outgroups (Galliformes, Crododylia, and Testudines) were also included. Nucleotide sequences were aligned, with amino acid alignment as a guide, using transAlign [28] and, for phylogenetic analyses, ambiguously aligned regions were removed using Gblocks 0.91 [29]. Phylogenetic reconstructions were performed using Bayesian inference (BI), using MrBayes 3.2.2 [30] and maximum likelihood (ML), using RAxML 8.0.20 [31]. Clade support on ML trees was evaluated by bootstrap re-sampling using 100 pseudo-replicates. The accession numbers for all sequences used in this work are shown in S1 Table.

### Selective pressure analyses

Identification of codons presenting evidence of positive selection was performed using the Bayes Empirical Bayes approach [32] as implemented in the CODEML program of PAML 4.7 [33]. Nonsynonymous/synonymous substitution ratios (ω), with ω = 1, < 1, or > 1 indicating respectively neutral, purifying, or positive selection, were calculated using both site models M7 and M8 [34], and a branch-site model [35]. In the branch-site analyses, two branch categories have been employed, namely cetacean versus non-cetacean lineages. Sites with probability P > 90% of belonging to the class with ω > 1 are inferred to be under positive selection.

### Protein structure analyses

In order to pinpoint the location of amino acids presenting signs of positive selection on AQP2, three-dimensional analysis of residue location has been performed by structure prediction by similarity modeling as implemented in SWISS-MODEL [36]. Previously published PDB (Protein Data Bank) entry 4oj2 presented the highest similarity to AQP2 and was used as template. Predicted structures were visualized and compared in SPDB Viewer [37].

## Results and Discussion

### Phylogenetics

**Fig 1:**
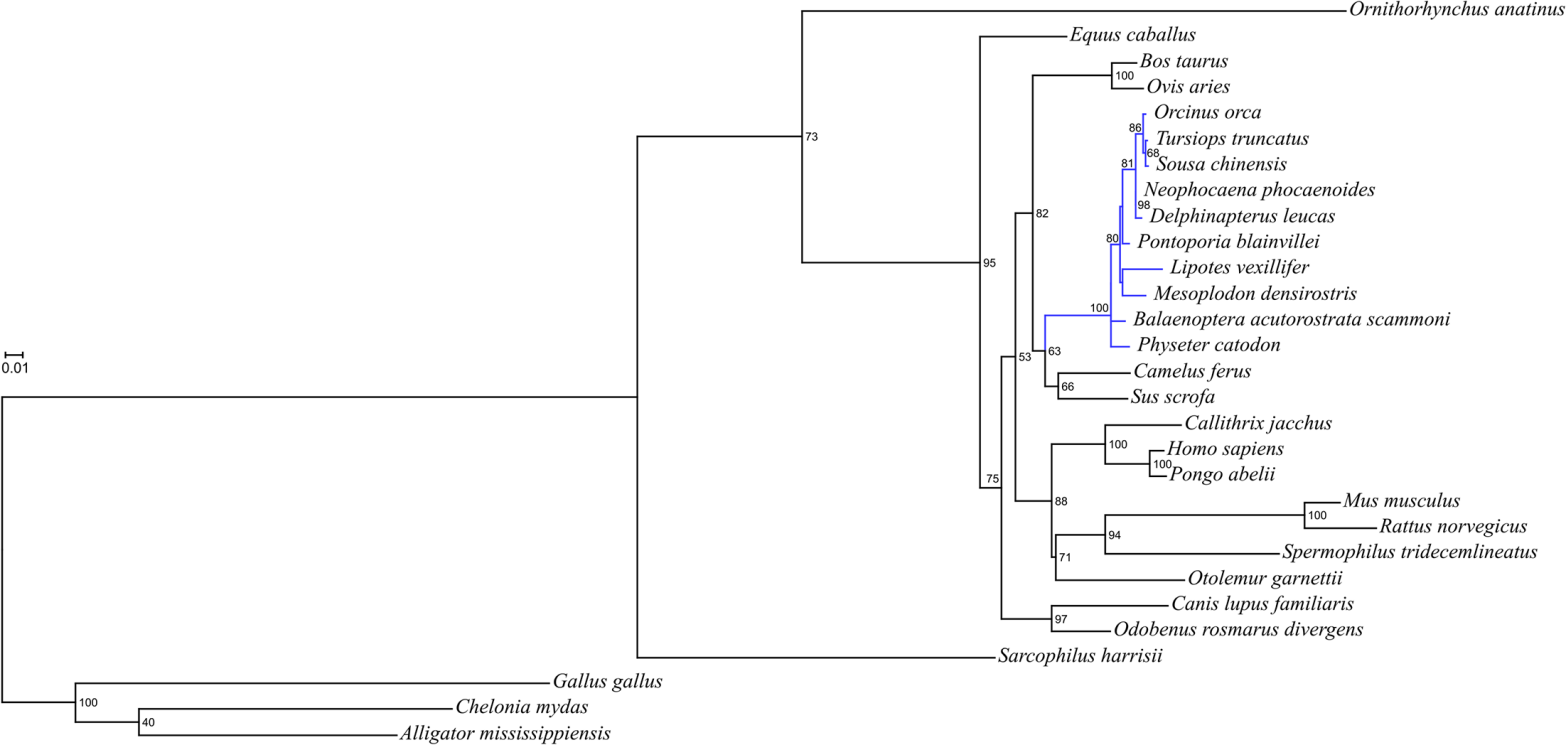
Phylogenetic tree using aquaporin 2 nucleotide sequences. The tree shows relationships among mammalian groups that are consistent with those derived from more comprehensive data sets. The tree was constructed using maximum likelihood, and clade support was evaluated by bootstrap re-sampling using 100 pseudo-replicates.

All aquaporin genes identified in the *P*. *blainvillei* genome were intact and there were no premature stop codons or frameshift mutations, which indicated the presence of functional proteins. Sequences from each aquaporin gene were used to construct phylogenetic trees using likelihood and Bayesian approaches. The topologies of the ML and Bayesian consensus trees were congruent, and the tree for AQP2 is shown as a representative example (Fig 1) due to its strong positive selection in cetacean-specific lineages (see below). In all AQP genes analyzed, most higher level relationships among mammalian orders and suborders were consistent with those of larger, more comprehensive data sets [6], in all AQP genes analyzed (S1 Fig).

### Detection of positive selection

A pair of site models (M7 vs M8) [34] was used to test for evidence of positive selection during the origin and diversification of cetaceans, in specific codons in the aquaporin genes. A small proportion of codons from AQP1 (codons 45 and 133), AQP4 (codon 74), AQP6 (codons 211 and 323), and AQP7 (codons 342, 343 and 356) were estimated to be under selection, with ω values of 1.36, 1.39, 2.11, and 2.45, respectively. In addition, all codons were identified by the BEB approach as having posterior probabilities ≥ 0.90.

**Fig 2:**
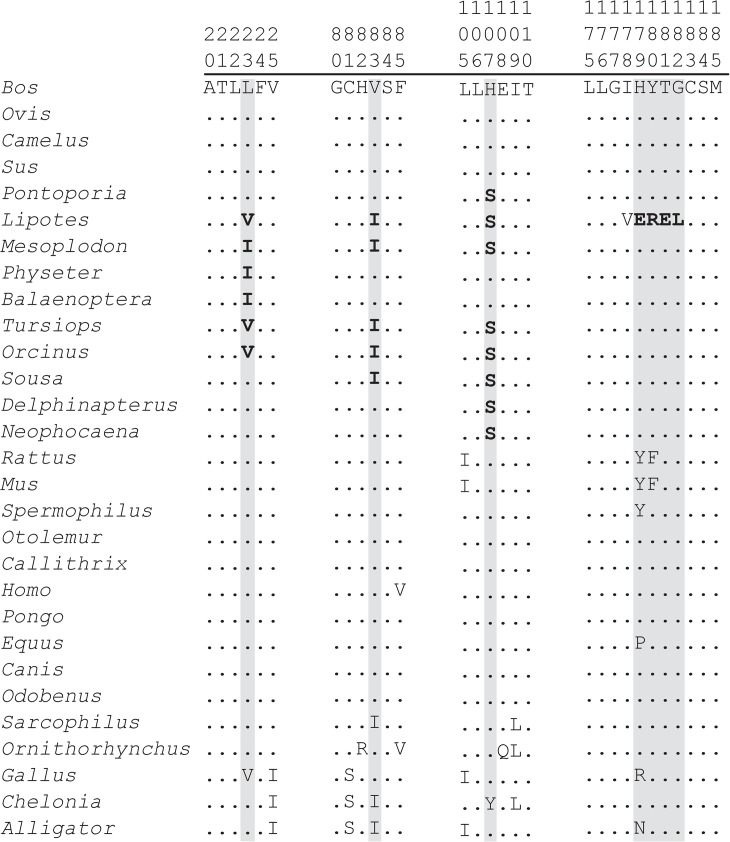
Partial alignment of mammalian AQP2 amino acid sequences. Dots represent residues that are identical to those on the first row of the alignment. A) Alignment showing amino acid substitutions in sites 23 (Leu → Val, Iso), 83 (Val → Iso), and 107 (Ser → His), occurring exclusively in the cetacean lineage. B) Alignment of sites 179, 180, 181, and 182 showing differences only in cetacean *Lipotes vexiliffer*, the only freshwater dolphin in our sample.

The branch site model was used to detect positive selection in a small number of sites that are sometimes hard to detect by the site model. The branch site constrained ω to be the same in the whole cetacean clade (ω1) and different for the rest of the mammals (ω0), using the LRT analysis. The selection analysis using the branch sites model suggested several positively selective sites, with seven identified in AQP2 (sites 23, 83, 107, 179, 180, 181, 182), five in AQP3 (20, 36, 103, 248, 256), one in AQP6 (27), and one in AQP9 (97) (Fig 2). These sites were detected only by this method, with overall probabilities greater than 0.9 using the Bayes empirical Bayes (BEB) test. However, the selection analysis using the branch site model showed that only one gene, namely AQP2, was subjected to strong positive selection exclusively in cetacean-specific lineages, whereas no selection was observed in terrestrial mammalian lineages.

**Fig 3:**
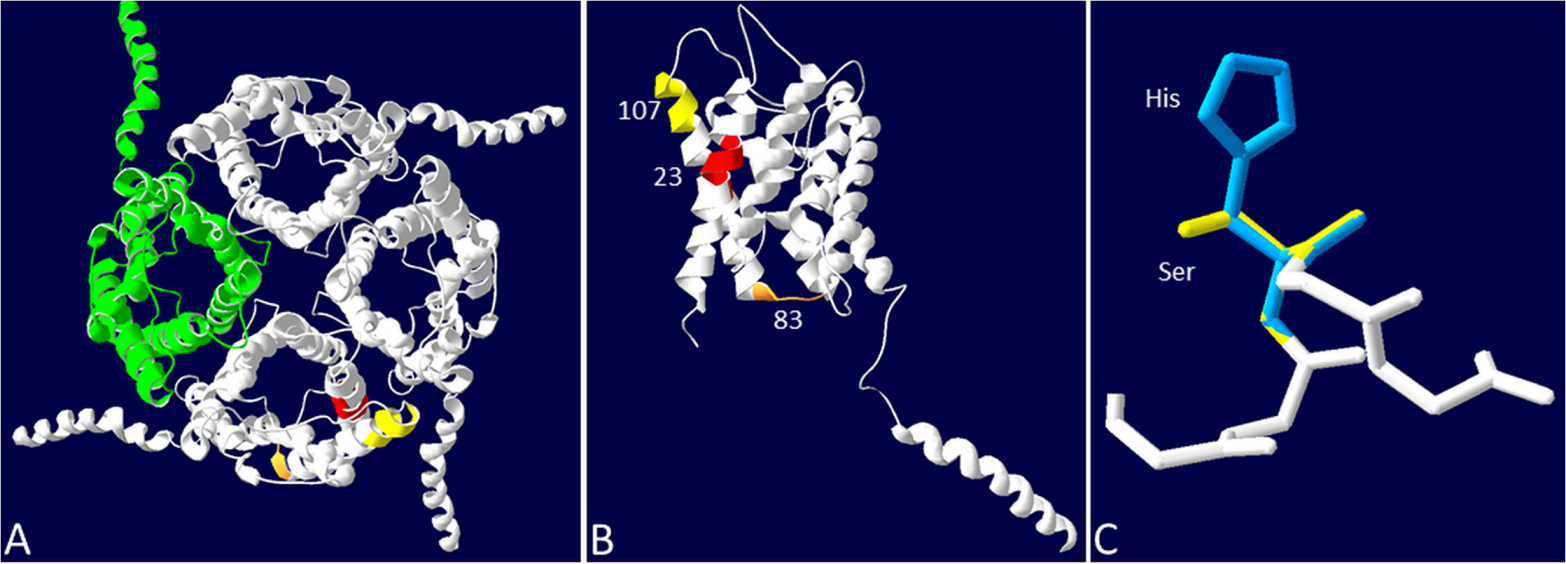
Three-dimensional mapping of positively selected AQP2 sites. A) AQP2 protein structure. Location of sites 23 (orange), 83 (red), and 107 (yellow) are highlighted. B) One subunit of AQP2 showing the location of sites 23, 83, and 107, which showed positive selection in cetaceans, colored as above. C) Superposition of the replacement of serine 107 by histidine, in the cetacean lineage.

Positively selected sites 23, 83, and 107 were located in, or close to, the functional regions in the predicted 3D structures of the AQP2 gene (Fig 3). For instance, residue 23 involves three hydrophobic amino acids (leucine, valine, isoleucine) among the sequences analyzed. The same pattern was observed at site 83, where valine and isoleucine were present. According to McClellan & McCracken [38], the amino acids valine, leucine, and isoleucine, when located in a transmembrane domain, as in AQP2, are more susceptible to substitution among each other because that results in conservative changes. On the other hand, two polar amino acids, histidine and serine, were observed at residue 107. As previously observed [39], serine and histidine are found in 81% of active sites. Site 107 was also found to be under strong positive selection by Xu et al. [12], where it was numbered as 105.

Signs of strong positive selection in AQP2 sites 179, 180, 181, and 182 were identified only in the baiji lineage. Similar results were also found in the work by Xu et al. [12] for genes ACE (angiotensin converting enzyme), AGT (angiotensinogen), and SLC14A2. According to Guo et al. [23] changes in osmoregulation strategies were found when observing urea serum levels in freshwater dolphins and their marine counterparts, being much higher in the former. Overall, the regulation of water permeability in the kidneys attributed to AQP2 is an important molecular strategy in cetaceans, suggesting an enhancement of their osmoregulation capacity. Clearly, AQP2 is required for production of concentrated urine by the kidney [19,20] and it has been found to be exclusively expressed in their collecting ducts [40,41]. This suggests that these freshwater dolphins may have distinct osmoregulation abilities to adapt to their divergent environments, or osmoregulatory adjustments related to different feeding habits [12,42].

Sites 179, 180, 181, and 182, are located near the conserved NPA (asparagine-proline-alanine) region of AQP2, and in a region of structural importance for the protein (see below). While the exact effect of each such substitution can only be devised by experimental approaches that are beyond the scope of the present study, the specific properties of the amino acids involved suggest that these changes could be important for protein function. Site 179 presented a histidine to glutamate substitution, which could be interesting for protein stability, since glutamate (negatively charged) tends to form salt-bridges with positively-charged amino acids. Also, glutamate has a higher tendency of being exposed to an aqueous environment, and being thus involved in substrate binding sites [43]. The substitution at site 180, tyrosine to arginine, could also have structural consequences, given the significant difference in side-chain characteristics between these two amino acids: tyrosine has a large, aromatic side-chain that is often involved in stacking interactions with other aromatic side-chains. Site 181 presented a threonine to glutamate substitution which, as mentioned above, could have significance in substrate-binding. And, finally, site 182 showed a substitution of glycine by leucine, which also involves very different amino acid side-chains: glycine is the simplest amino acid, with a side-chain of a single hydrogen atom, which allows for much more conformational flexibility in the peptide chain. Leucine, on the other hand, has a preference for being buried in hydrophobic environments, thus being much more often found in alpha helices than in beta strands. Glycine, due to its side-chain's properties, can also play a distinctive functional role, for example by allowing the binding of phosphate. Leucine's side-chain, on the other hand, is very non-reactive and is rarely involved in protein function. Since our results showed strong selective pressure, on positions 179–182, on the river dolphin lineage only, more studies focused on other cetaceans adapted to fresh water environments are needed, especially considering that present evidence indicates that they adapted to riverine environments independently [44,45]. If similar changes were observed in these other freshwater species, it would support the marked importance of the AQP2 gene in osmoregulation capacity, and its importance for survival in that environment.

Our results suggest that the adaptive evolution in the AQP2 gene occurred during the origin and diversification of cetaceans. Our results have shown positively selected sites in AQP1 (45), AQP4 (74), and AQP7 (342, 343, 356) both in the Artiodactyla and Cetacea lineages, suggesting these events are not related to maintenance of water and electrolyte homeostasis in seawater.

### Spatial distribution of the positively selected sites in the 3D structures

A set of six AQP2 sequences from a number of organisms (*Balaenoptera acutorostrata scammoni*, *Bos taurus*, *Lipotes vexillifer*, *Physeter catodon*, *Pontoporia blainvilei*, and *Tursiops truncatus*), representing the diversity of residues under positive selection, were used for structure prediction. The quality of predicted models were estimated by Global Model Quality Estimation (GMQE), which also considers QMEAN [46] to increase the reliability of quality estimation. High GMQE values were observed ranging from 0.83 to 0.91 (data not shown), indicating a good modeling of AQP2. Fig 3 shows the complete model of *P*. *blainvillei*’s AQP2, composed by four protein subunits and the same number of pores, as revealed by electron microscopy by Vahedi-Faridi et al. [47]. Although a good prediction was obtained and substitutions were present at specific sites, the structural differences among the structures were minimal. For example, using SPDB Viewer [37] to compare the spatial position of all 253 alpha-carbons between the predicted structures of *Bos taurus* and *Tursiops truncatus* resulted in only 0.02Å difference (data not shown).

The *P*. *blainvillei* AQP2 model illustrates the location of the positively selected sites (23, 83, 107) among Cetaceans (Fig 3B). All of them were located at the pore and may play an important role in water transport [48]. However, as mentioned previously, sites 23 and 83 were related to substitutions among similar hydrophobic amino acids, which may present comparable function [38]. On the other hand, residue 107 (Fig 3B) resulted in substitution of a positively charged amino acid (histidine) to an uncharged one (serine). In this case, the lack of the aromatic ring in histidine could influence interactions among the neighboring amino acids, as already observed in other molecules [49].

Sites 179 through 182, which were found to be positively selected exclusively in the baiji lineage, are located right inside the pore, in the transition between a short helix and a loop (data not shown). Although the understanding of the exact significance of these positively selected changes would require direct experimental data on their exact structural and functional effects, it is suggestive that they have occurred in a possibly crucial portion of the molecule. This leads to the speculation that these changes could be related to the freshwater environment in which this specific dolphin lineage lives.

## Conclusions

Aquaporins are membrane proteins present in all forms of life with the main function of osmoregulation. In order to try to clarify what changes have occurred in the genes of aquaporins during the passage of cetaceans from the terrestrial environment to the aquatic, the present study analyzed the molecular evolution of aquaporin genes. Our analyses reveal strong selective pressure only in the AQP2 gene, exclusively involving the cetacean lineage. The AQP2 is the only aquaporin found exclusively in the kidneys and involved in the reabsorption of water by collecting tubules, being clearly required for production of concentrated urine–a crucial osmoregulation mechanism in cetaceans.

Analyses of the 3D protein structures have not revealed significant structural differences. It is worth noting however that site 107, which is positively selected in cetaceans, is positioned very close to the pore. This could be affecting water collection function in the duct and should be tested in the future. This suggests adaptive changes in the evolutionary process in response to environmental osmotic conditions. The analysis also revealed strong selective pressure on positions 179 to 182 (also positioned in a functionally important part of the protein) of gene AQP2 on the only river dolphin lineage present in this study, therefore more studies should be focused on this and other freshwater species of cetaceans to further elucidate the importance of AQP2 in osmoregulation capacity, and its role for survival in a osmotically challenging environments.

## Supporting Information

S1 Fig Maximum likelihood phylogenetic trees inferred using nucleotide sequences from aquaporins AQP1, AQP3, AQP4, AQP6, AQP7, and AQP9. Branches in blue indicate the cetacean lineages. Clade support was evaluated by bootstrap re-sampling using 100 pseudo-replicates (only values of 50 and above shown).
(JPG)

S1 Table Nucleotide sequences of aquaporins used in this study, including taxonomy and access numbers in the NCBI database. (JPG)
