# Supplementary figures and images for: Evidence of Positive Selection of Aquaporins Genes from Pontoporia blainvillei during the Evolutionary Process of Cetaceans
Source: PLoS One. 2015 Jul 30;10(7):e0134516. doi: 10.1371/journal.pone.0134516 (PMC4520692; doi:10.1371/journal.pone.0134516)

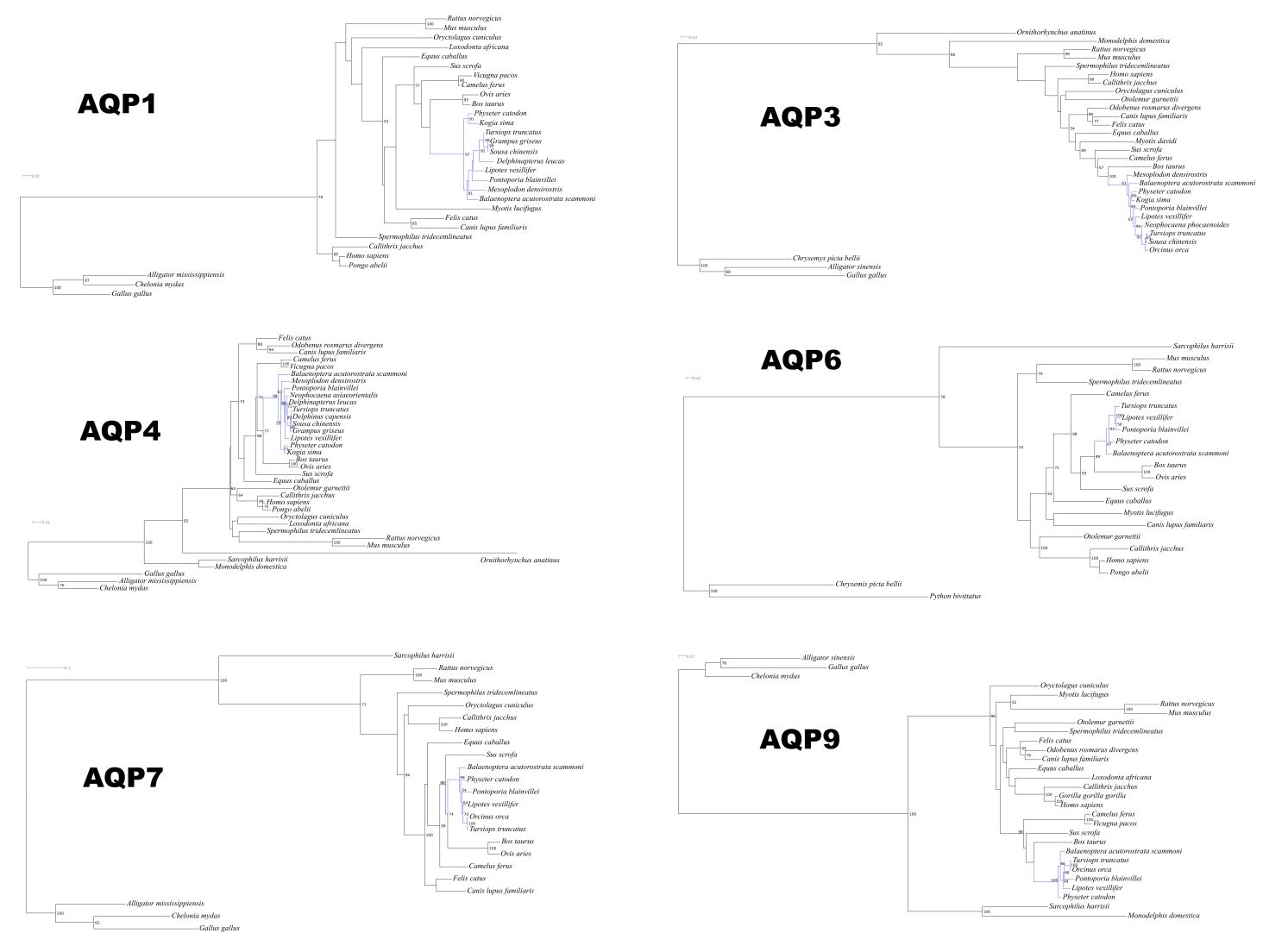

Supplement: S1 Fig — Branches in blue indicate the cetacean lineages. Clade support was evaluated by bootstrap re-sampling using 100 pseudo-replicates (only values of 50 and above shown). (JPG) [file pone.0134516.s001.jpg]

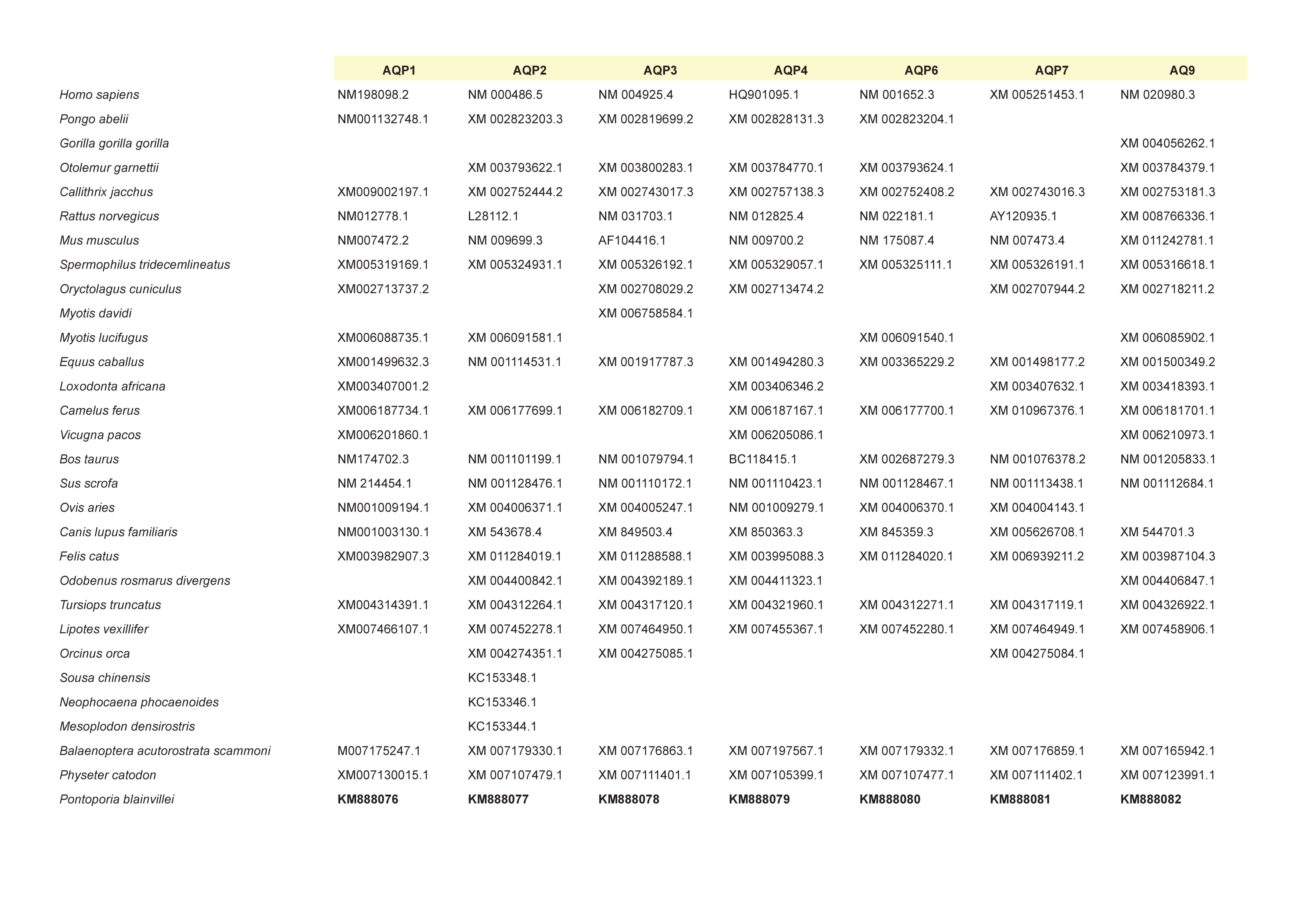

Supplement: S1 Table — (JPG) [file pone.0134516.s002.jpg]
